# Barriers to postpartum health and opinions on a postpartum peer navigator program amongst refugee women resettled in California

**DOI:** 10.1186/s12884-025-07479-2

**Published:** 2025-03-29

**Authors:** Sarah Alsamman, Ruth Teseyem Tadesse, Khatira Sarferaz, Amina Sheik Mohamed, Sheila K. Mody

**Affiliations:** 1 School of Medicine, University of California, San Diego. La Jolla, CA USA; 2 Altman Clinical and Translational Research Institute, Center for Community Health-Refugee Health Unit, University of California, San Diego. La Jolla, CA USA; 3 Department of Obstetrics, Gynecology and Reproductive Sciences, OB Refugee/Asylee Care Navigator Program, University of California, San Diego. La Jolla, CA USA; 4 Division of Complex Family Planning, Department of Obstetrics, Gynecology and Reproductive Sciences, University of California, San Diego. La Jolla, CA USA

**Keywords:** Postpartum, Refugee, Peer navigator

## Abstract

**Background:**

The aim of this study is to identify postpartum challenges and assess for interest in a postpartum peer navigator program amongst refugee women resettled in California.

**Methods:**

Participants were recruited through ethnic community-based organizations in California using convenience and snowball sampling. Arabic, Dari, or Pashto speaking women who have given birth in the United States within the last five years and entered the country as refugees, special immigrant visa holders, or asylum seekers were eligible to participate. Semi-structured interviews were analyzed using Braun and Clarke’s thematic analysis.

**Results:**

We interviewed 26 participants. The mean age was 30 years (SD = 6.3) and mean length of time in the United States was 4.5 years (SD = 3.0). All participants are state insurance recipients. Most participants (89%, n = 23) utilize an interpreter. We identified seven themes: (1) lack of comprehensible postpartum information; (2) displacement and isolation worsen postpartum mental health; (3) stigma and fear discourage seeking postpartum mental health care; (4) barriers in interpretation undermine postpartum care recommendations; (5) interest in a language concordant postpartum navigator.

**Conclusion:**

Refugee women encounter challenges with contraception and mental health care postpartum exacerbated by language barriers and difficulties with interpreter use. There is interest in language concordant postpartum peer navigation as tool to mitigating these challenges.

**Supplementary Information:**

The online version contains supplementary material available at 10.1186/s12884-025-07479-2.

## Introduction

Postpartum care is critical for maternal mental and physical health as well as a bridge to long-term engagement with the health system. However, 30% of women in the United States (US) do not receive postpartum care [1]. This gap in care is an important opportunity to improve maternal care. In 2018, the American College of Obstetricians and Gynecologists published guidance recommending that postpartum care be “an ongoing process, rather than a single encounter” and “tailored to each woman’s individual needs” [2]. Efforts to customize postpartum care, increase postpartum visit attendance, and ensure adherence to best practice recommendations would be beneficial.

Studies indicate that Medicaid populations, those with low socioeconomic status, and recent immigrant/refugee populations face barriers to postpartum care [3, 4]. Patients who have been forcibly displaced are at particularly increased risk for maternal morbidity and postpartum complications due to unique stressors experienced both pre- migration and post-migration during resettlement [5–8]. Ultimately this perpetuates the health disparities that exist in refugee communities. Refugee women are at higher risk for developing postpartum diabetes and are also more likely to experience postpartum depression, with an increased likelihood of visiting the emergency department for a mental health concern postpartum [9, 10]. Refugee women resettled in high income countries are less likely to attend postpartum visits than women born in the host country [11]. The barriers to accessing care include language, technology, and limited understanding of the healthcare system. Postpartum care is a missed opportunity to improve long-term maternal health in this population.

Postpartum peer navigation is an effective tool for improving postpartum outcomes.

Navigating New Motherhood, a postpartum patient navigator program based at a university tertiary care center’s hospital-based women’s health clinic, documents increased retention in postpartum care and frequency of contraception uptake, depression screening, and vaccination in a community of low-income women receiving support from a navigator [12, 13]. However, the barriers that refugee women face in interacting with the healthcare system may persist in the peer navigation model. Multiple studies over the past two decades demonstrate differences in provider medical decision making by patient race due to unconscious or implicit biases [14]. Recent studies have been conducted showing racial concordance increases perceived respect and trust in their care provider [15–18]. However, to our knowledge, no studies have directly engaged refugee women on the type of postpartum support they perceive as useful and the needs they need addressed through such a program. This study explores the postpartum experiences of refugee women and assess their interest in and opinions on a postpartum peer navigator program. We suspect that a language and ethnic concordant postpartum peer navigator program will be perceived as a tool that can overcome these barriers and improve postpartum care utilization, maternal experiences, and maternal outcomes.

## Methods

### Study design

We conducted a descriptive qualitative study utilizing semi-structured one-on-one interviews. The interviews included questions exploring different domains of postpartum health including mental health, breastfeeding, and contraception (see Additional file 1). Participants were also asked demographic questions about their country of origin, insurance status, and other related topics. Questions also assessed interest in a postpartum navigator program. The interview guide was developed with input from organization leaders familiar with community being sampled from and was pilot tested before use. All participants were offered an in-person or virtual interview, but all chose a virtual interview. The study was approved by the University of California, San Diego Institutional Review Board and adhered to the Consolidated Criteria for Reporting Qualitative Research (COREQ) checklist (see Additional file 2).

### Sampling and recruitment

Participants were recruited between August and December 2023 using convenience and snowballing sampling through community organizations in Sacramento and San Diego, the top two sites for refugee resettlement in California [19]. Participants were approached via a phone call from a research team member in which the study aims, eligibility criteria, and participation requirements were shared. The eligibility for participation included reproductive age females 18 years or older who resettled in the US as refugees, special immigrant visa holders, or asylum seekers and delivered a baby in the US within the last five years. Eligible participants were then scheduled for an interview.

### Data collection

Interviews lasted approximately 45 min and were audio recorded. Two bilingual female research team members, a medical student researcher (SA) and community health worker (KS), both with prior experience in interviewing and working with refugees conducted all interviews virtually in the participants’ primary language. Researchers did not have a personal relationship with the participants before commencement of the study. At the beginning of each interview the facilitating researcher introduced herself, including her name and occupation, and described the overall aim of the study. No other individuals were present during the interview besides the researcher and the participant. Participants were compensated with a $50 gift card. We conducted interviews until thematic saturation was achieved and new ideas were no longer obtained from additional interviews. After each set of five interviews, transcripts were reviewed to assess whether new insights were still emerging. Once multiple consecutive interviews yielded recurring themes without introducing substantively new concepts, the team agreed that saturation was reached, and data collection was concluded. No repeat interviews were conducted.

### Analysis

Two bilingual research team members, SA and KS, translated and transcribed interviews from Arabic, Dari, or Pashto to English. Translators were of Arab and Afghan decent with native fluency in Arabic or Dari/Pashto and a deeply rooted understanding of the study population’s cultures. Two members of the research team, SA and RT, conducted inductive thematic analysis utilizing Braun and Clarke’s method with Dedoose, a qualitative data analysis software [20, 21]. Specifically, SA and RT read all the transcripts to familiarize themselves with the data. After prolonged engagement with the transcripts, a codebook was developed based on recurring motifs. Two research team members coded all interviews independently and then met to compare codes and resolve all coding discrepancies. A list of recurring themes that emerged in the analysis was developed with a third research team member, SM, through iterative reflection. Themes were consolidated and the team developed a final interpretation of the data that accurately represented the interviews. Trustworthiness of the data was obtained through credibility, transferability, dependability, and confirmability [22, 23]. Credibility was established through prolonged engagement with study participants and interview transcripts, as well as an interdisciplinary, multi-cultural research team with both lived and professional experience in refugee health that provided different perspectives, promoting research triangulation. Thorough description of the sampling method, participant eligibility criteria, research context and data collection methods ensure transferability. Dependability was attained through methodological documentation and maintenance of an audit trail. Lastly, confirmability was obtained through peer debriefing by engaging with the San Diego Refugee Forum, a network of experts in refugee health outside the research team, to obtain feedback on the themes and associated interpretation. Forum members, many of whom have lived experience with displacement, provided alternative perspectives on the data and confirmed the relevance of our findings.

## Results

### Study participants

A total of 26 women were interviewed. All enrolled participants completed the study and received compensation. The average age of participants was 30 years (SD = 6.3) and the mean number of years resettled in the US across our sample was 4.5 years (SD = 3.0). With respect to primary language, 53.8% (*n* = 14) of participants spoke Arabic, 34.6% (*n* = 9) spoke Dari, and 11.5% (*n* = 3) spoke Pashto, and a majority of participants used an interpreter in healthcare settings (88.5%, *n* = 23). All participants were publicly insured. Most participants last delivered within two years of being interviewed (73.1%, *n* = 19). Only 15.4% (*n* = 4) of participants were first time mothers. A small number of participants experienced a pregnancy complication, such as diabetes or hypertension (30.8%, *n* = 8). Participant demographic information is further summarized in Table 1.

**Table 1:** Demographic characteristics of participants (*n* = 26)

**Country of origin**	N value or mean (percentage or standard deviation)
Syria	13 (50.0%)
Afghanistan	12 (46.2%)
Sudan	1 (3.8%)
**Language spoken at home**	
Arabic	14 (53.8%)
Dari	9 (34.6%)
Pashto	3 (11.5%)
**Age (years)**	
Mean	30 (6.3)
**Years in the United States**	
0–1 years	4 (15.4%)
2–3 years	8 (30.8%)
4–5 years	3 (11.5%)
6–7 years	7 (26.9%)
8 + years	4 (15.4%)
**Highest level of education**	
Preliterate	2 (7.7%)
Primary School	8 (30.8%)
High School	13 (50.0%)
College Degree	3 (11.5%)
**Marital Status**	
Married	26 (100.0%)
**Interpreter use**	
Yes	23 (88.5%)
No	3 (11.5%)
**Health insurance**	
Public	26 (100%)
**Time since last delivery**	
Within the last year	9 (34.6%)
1–2 years ago	10 (38.5%)
2–3 years ago	4 (15.4%)
3–4 years ago	1 (3.8%)
4–5 years ago	2 (7.7%)
**First time mom**	
Yes	5 (19.2%)
No	21 (80.8%)
**Breastfed after last delivery**	
Yes	23 (88.5%)
No	3 (11.5%)
**Pregnancy complication**	
High blood pressure	6 (23.1%)
Gestational diabetes	2 (7.7%)
None	18 (69.2%)

### Themes

The following five themes emerged from the data: (1) lack of comprehensible postpartum information; (2) displacement and isolation worsen postpartum mental health; (3) stigma and fear discourage seeking postpartum mental health care; (4) barriers in interpretation undermine postpartum care recommendations; (5) interest in a language concordant postpartum navigator.

#### Theme 1: Lack of comprehensible postpartum information

Participants reported not receiving enough information or not understanding the information that they received about their delivery experience and various aspects of postpartum care. Many participants relied on their prior experiences and cultural practices to guide them postpartum. First time mothers especially expressed having many questions postpartum and reliance on family and friends for information. Many participants, regardless of previous experience, highlighted having limited information on navigating the healthcare system during pregnancy and postpartum.

*“Yes, I still have questions about how and when to take the medication I was prescribed postpartum. I asked my young son to read the discharge papers for me.”* (Afghanistan, Age 30).

##### Inaccessible postpartum contraception counseling

Participants particularly emphasized a gap in information on postpartum contraception options from their clinician, which left them unsure of how to obtain contraception. Participants that received counseling reported that it was too complex or not language accessible and they remained uncertain of which type to use or had unanswered questions.

*“I didn’t know where to get them [contraception] from or where to find them. We were very lost in the beginning. Life in America is not easy.”* (Afghanistan, Age 30).

*“Well, I was the first one who came here [to the US] and became knowledgeable about contraception. At the clinic, they tried to explain to me, but I didn't understand much because of the language. So, I tried to look on Google to learn about what the implant was and what the injections were. My mom and in general back home, we don’t really know what an IUD is. These things are all new, not from their [our parents] time.”* (Syria, Age 28).

##### Reliance on informal sources for postpartum information

Participants turned to family members or friends for information about postpartum contraception and other postpartum care topics. For some participants, information from family or friends even superseded that from clinicians. However, from these sources they often received inaccurate information. For example, multiple participants shared inaccurate information they received from family or friends about the impact of hormonal contraception methods on fertility, mental health, and breastfeeding which discouraged them from the use of these methods.

*“When the doctor assured me that the injection does not impact [fertility] I was convinced by it. After I got the injection, the ladies around me asked, ‘What type of contraception did you get?’ When I told them I used the injection; they all discouraged me from it. So—I did not go get it anymore.”* (Syria, Age 27).

*“I used the arm implant, and I only had my period once during the past year. I have also been experiencing many mood fluctuations. Many people have advised me to remove the implant because it is not healthy.”* (Afghanistan, Age 28).

Participants also searched online in their primary language for more information. They used generic search engines and did not report using a particular online source consistently.

##### Desire for language accessible postpartum resources

In general, there was a desire for more language-accessible and printed information about postpartum contraception and other aspect of postpartum care. Some reported receiving printed information in English at a postpartum visit, which they were unable to use.

*“We searched on Google, and my husband read for me. We also watched videos, and my husband translated them for me. It would have been better if the paper [from my obstetrician] was translated into Dari.”* (Afghanistan, Age 41).

“*I wish she [my obstetrician] gave me a paper [about contraception options]. I would have read about them at home and really understood them.”* (Syria, Age 19).

#### Theme 2: Displacement and isolation worsen postpartum mental health

##### Emotional burden of displacement

Participants described how the stress of having a newborn exacerbated the existing stress of being separated from family, adjusting to a new country, and coping with forced displacement. Participants felt that the traumatic experience of displacement and the stress of resettlement contributed to their postnatal emotional distress. This is because these women not only had to cope with the arrival of their newborns but also with the loss of their homeland and family at the same time.

*“I cried every day. I am stressed. I am in a foreign country. I fled a country in the midst of war and there were bombs being dropped on us. I am worried about my newborn son. I am worried about my daughters. I am worried about my life. This [emotional reaction] is something normal that people experience after going through all the things that I went through. My body has not even acclimated to the US, and so of course I had postpartum depression.”* (Syria, Age 41).

*“This makes a person live in depression, because it is very hard. You long for family…. you long for everything to be as it was. However, everything has changed. So, this really impacts your mental state.”* (Syria, Age, 35).

##### Postpartum loneliness and lack of traditional support systems

Participants cited forced separation from their families as one of the largest challenges they faced postpartum and described lack of social support as a precipitating factor for mental health challenges after delivery. For example, one participant described delivering and caring for her newborn along with her other children completely on her own after being forcibly separated from her husband who remains in Afghanistan. Many described feelings of loneliness, exhaustion, and anxiety, exacerbated by the absence of traditional support structures they had in their home countries.

*“There is a saying in my culture that says an individual always feels better after speaking to another person. However, it was very difficult since I was all alone here with my two kids and my husband was in Afghanistan.”* (Afghanistan, Age 39).

*“The hardest part [of my postpartum experience] was the absence of my mom. I was alone.”* (Syria, Age 28).

Participants shared a longing for the presence and assistance of their family members, particularly their mothers, postpartum. Many contrasted their experience in their home countries, describing an absence of their key support systems in the US. Participants described cultural customs that they practiced postpartum in their home countries but were not able to practice in the US due to the absence of family. In their home countries the birth of a newborn brings the family together as mothers, sisters, and aunts gather around the new mother to support her.

*“In Afghanistan, when my daughter was born, I stayed in the hospital for one day and then went home. I had family at home to take care of me. For 25 days they did everything for me and assisted me with the baby. Here I stayed in the hospital for five days and only my husband was with me. After I went home, I had to do everything myself even though I had a cesarean section.”* (Afghanistan, Age 30).

*“Being far from family is very difficult. At a time like this a person needs their family around them to help them and support their mental health. In Sudan the customs [postpartum] are very different. The woman spends 40 days with her mother who helps her with everything and prepares specific foods for her. Here I did not have this support because my mom is not with me.”* (Sudan, Age 22).

#### Theme 3: Stigma and fear discourage seeking postpartum mental health care

##### Cultural stigma

Participants expressed apprehension about seeking help or discussing mental health concerns with clinicians. Participants pointed to differences in cultural understanding of mental health and a desire to maintain family privacy. They perceived western approaches to mental health care as unuseful and perceived to be overcomplicated. Additionally, participants minimized their postpartum mental health problems to justify not seeking care.

*“No, I preferred that this remain between me and my family. It is not a topic to discuss with doctors. You know how their thinking is here…it is different than how we think.”* (Syria, Age 19).

*“If I told them I was depressed, they would start investigating and asking more questions. For me, the concept of postpartum depression is not a big deal. I was aware of it and knew what to expect. [When I experienced it] I treated myself.”* (Syria, Age 28).

##### Avoiding mental health care due to fear of being deemed inadequate

Some participants, despite experiencing symptoms of postpartum depression, avoided seeking mental health care out of fear of being deemed incapable of caring for their children. They expressed distrust in the healthcare system and believed that there would be systemic repercussions to obtaining mental health support.

*“I must be cautious of what I say about my feelings because I don't want them to think I have any psychological problems. […] I had a lot of things going on postpartum—I was in pain, I was stressed, and I could not pray for 16 days. I felt like I was going to explode. I just wanted to cry, but I was afraid that they would take my son away from me. So, I did not talk about my feelings at all.”* (Syria, Age 41).

#### Theme 4: Barriers in interpretation undermine postpartum care recommendations

##### Interpreter challenges

Most participants reported that language and difficulties with using an interpreter were key issues contributing to their postpartum health problems. Participants did not feel that interpreters relayed all pertinent information during their healthcare encounters. Participants perceived interpreters to be shortening their statements or incorrectly interpreting them. Technical and audio difficulties were also cited as barriers to communicating through an interpreter. Even when participants felt that the interpreter understood them, they ultimately did not feel understood by the clinician.

*“The hardest thing is the language barrier. Sometimes when I want to express how I feel, even though the interpreter understands me, they do not interpret what I am saying correctly or do not get the right point across. Therefore, I find it very hard to get my point across to the doctors.”* Syria, Age 41).

Furthermore, participants shared experiences of being provided with an interpreter that speaks a different dialect limiting their ability to communicate. Thus, they preferred to turn to family members to interpret.

*“The interpreter does not always accurately explain the point that you are attempting to get across to the doctor. Sometimes they speak a different Arabic dialect making it difficult to communicate the exact point that you are trying to make.”* (Syria, Age 30).

*“I feel more comfortable when my husband is interpreting for me. Also, a lot of the interpreters only speak Farsi, so we do not understand them very well.”* (Afghanistan, Age 30).

Finally, participants were uncomfortable when provided a male interpreter in the postpartum setting and they tended to restrict the information they shared with clinicians when communicating through male interpreters. Participants did not feel comfortable asking for a different interpreter or were uncertain if they were permitted to ask.

*“I experienced difficulties with the interpreter when I went to the obstetrician. When I asked for an interpreter, they were all male. I felt shy and not know how to talk to him. I was also not able to tell the interpreter that I wanted a female interpreter. I found this very difficult.”* (Syria, Age 35).

##### Language barriers undermine postpartum care

Many participants describe misunderstanding or not understanding postpartum care recommendations because of these communication challenges. Misinterpretation led to confusion and anxiety about their medical conditions and treatments. For example, one participant was incorrectly informed that she had cervical cancer, which caused significant stress and long-term distrust of interpreter services.

*“They conducted a pap smear, but the interpreter misinterpreted. Instead of saying that the test was to screen for cervical cancer, they said I had cancer. I told my husband and family that I had cervical cancer, and we were very worried and sad. Then, a while later, when we were informed the pap smear results were negative, my husband and I were very confused.”* (Afghanistan, Age 28).

##### Preference for language concordant clinicians

Many participants who received care from an English-speaking obstetrician for their first pregnancy in the US sought out obstetricians who spoke their primary language for their subsequent pregnancy. They expressed a sense of comfort, ease, and trust when interacting with a clinician who shared their cultural heritage. Some participants felt that language concordant clinic staff made scheduling appointments easier and thus improved access to postpartum care.

*“I prefer an Arab doctor. This is because even if though I speak English fairly well there is still a roadblock, especially while giving birth.”* (Syria, Age 38).

*“My doctor currently is from Afghanistan, and I can share everything with her and speak freely.”* (Afghanistan, Age 31).

#### Theme 5: Interest in a language concordant postpartum navigator

All participants expressed strong interest in a postpartum navigator program. There was a desire for a point person that provides informational support postpartum to assist in understanding complicated healthcare and social service networks. Participants did not know how to engage with the healthcare system after giving birth in the US and noted that a navigator could help clarify typical postpartum care processes. Furthermore, participants identified navigators as particularly useful for mothers with postpartum depression or other postpartum complications.

*“I think it would be useful to have a program like this, especially for inexperienced mothers like me. For instance, I did not know that I could visit the doctor postpartum. This would help us a lot.”* (Afghanistan, Age 28).

*“When we first arrive in the US, we don’t have any language skills, but we have a lot of questions. Especially for mothers who have postpartum depression, there needs to be someone present to whom we can ask questions. We need someone to explain to us what happens here after giving birth.”* (Syria, Age 35).

##### Navigator preferences

Participants were not interested in a navigator who did not speak their primary language, noting that it would not be much more helpful than the support available to them currently. All participants thought that the postpartum navigator should be language concordant for clearer communication, efficiency, and stronger rapport.

*“Yes, it is necessary that the navigator speaks the same language as the mother so that they can communicate. If the navigator does not speak the same language, then what is the difference [from existing resources].”* (Syria, Age 22).

Some participants thought that navigator ethnic concordance would foster comfort and trust, especially when it came to religious practices or preferences. Others thought that ethnic concordance was not important, citing language concordance as far more important.

*“Life is already hard so we don't want to add someone who would make it more stressful. […] We need support from someone who has the same traditions and practices as us so that we can feel comfortable with them and not have to explain ourselves.”* (Syria, Age 41).

*“As long as the navigator speaks the same language, their country of origin and background doesn’t matter.”* (Afghanistan, Age 26).

All participants expressed a preference for female navigators. They emphasized the need for a female navigator to feel comfortable openly discussing their reproductive health concerns.

*“The most important thing is that the navigator is female, not male. So, there is no discomfort when an individual talks to them about certain topics.”* (Syria, Age 26).

## Discussion

This qualitative study used Braun and Clarke’s thematic analysis to examine the postpartum experiences of 26 refugee women resettled in California. We identified several barriers to postpartum health in this sample including lack of social support, stigmatization of mental health care, misinformation on postpartum contraception, and difficulties with interpreter use. Additionally, we characterized interest in and opinions on a postpartum navigator program as a tool to overcoming these challenges.

One of main results of our study was that many participants report not receiving or not understanding postpartum contraceptive counseling. This finding aligns with previous studies conducted in high income countries which demonstrate that refugees are less likely to receive contraceptive counseling and are less likely to be prescribed a contraceptive method [24–26]. Subsequently, refugee women are more likely to experience unwanted pregnancies [26]. Our study confirms that the gap in understandable contraceptive counseling persists in the postpartum setting and may be due to difficulties with interpreter use. Factors described by participants such as distrust of information received through an interpreter and minimizing the number of questions asked during clinical encounters with an interpreter may contribute to the gaps in postpartum contraception understanding. Several participants shared concerns about the impact of specific hormonal contraception methods on future fertility, mood, and breastfeeding and described receiving this information from friends or family members. The presence of inaccurate information in the community influenced postpartum family planning, despite negating information received from clinicians. These findings are consistent with prior studies among other refugee communities that report fear of hormonal methods across cultural groups [27, 28].

Another major finding of this study was the lack of social support and isolation experienced postpartum among refugee women. Consistent with existing literature on the higher rates of postpartum depression amongst refugee mothers, participants in our study shared experiences of poor mental health postpartum, exacerbated by feeling lonely, longing for family and homeland [7, 29, 30]. Some participants in our study expressed discomfort accessing care out of fear of being deemed an incapable mother and facing systemic consequences. Similarly, a study of immigrants in Canada identified fear about losing custody of their children as a barrier to accessing postpartum mental health care [31]. Additionally, congruent with other research, participants shared lack of faith in western medicine approaches, desire for privacy and language barriers as key factors in shaping help-seeking behavior [32]. Many women in our sample normalized postpartum depression and acknowledged its reality even if they had not experienced it themselves which indicates adequate awareness of postpartum depression.

Limited English proficiency is well-documented as a predictor of poorer health outcomes [33]. Most of our participants endorsed attending at least one postpartum care visit but reported that language was a key barrier to understanding the recommendations made at those visits. Consistent with existing literature, our participants described distrust of interpreters, discomfort with male interpreters, and patient-interpreter dialect differences as persistent drivers of communication challenges [34–36]. For our study participants, these challenges resulted in misunderstanding of postpartum diagnoses and instructions. While discussing the postpartum navigator program many participants suggested that navigators also serve as interpreters to help overcome some of the challenges they faced with interpretation. Although the role of interpreters is typically to solely provide verbatim translation some studies demonstrate a desire amongst providers and patients for interpreters to act as cultural mediators [37]. An interpreter/doula program at a large urban maternity center in the US that trained certified Spanish interpreters in labor and postpartum doula skills documented increased patient and staff satisfaction [38]. Thus, language concordant postpartum navigation may be an effective tool in alleviating communication limitations in the postpartum healthcare setting.

Participants in our study demonstrated a strong interest in a navigator program. Our study is one of the first to characterize the opinions of refugee mothers on a postpartum navigator program. The roles of a patient navigator have been recommended in previous research and include identifying and mitigating individual barriers to care access, improving the timeliness of care, providing health education, as well as offering social support [39, 40]. Our participants were most interested in a navigator who could offer information on the healthcare system in the US and provide support in scheduling postpartum appointments, securing transportation to appointments, and completing documentation associated with having a newborn (e.g., birth certificate and social security card). They also desired a navigator they could ask basic questions about breastfeeding or contraception. This finding is consistent with another study evaluating an existing postpartum navigator program, in which providers reported a desire for navigators trained in basic postpartum health education [41]. Postpartum navigators may have an impactful role on the postpartum contraception knowledge gaps we identified in our study. Furthermore, participants suggested that the navigator be trained in providing emotional support and identified mothers experiencing postpartum depression as key beneficiaries of such a program.

All participants in our study urged that the navigator should be language concordant citing reasons of efficiency, trust, and understanding. Participants did not think that the navigator needed to be ethnically concordant. However, multiple studies report that racial concordance in healthcare encounters increases the perceived trust of the healthcare providers [15–18]. Social desirability bias may have played a role in our findings as participants could fear being perceived as discriminatory or prejudiced if they expressed a preference for an ethnically concordant navigator. Language concordance was identified as a key factor in building trust and rapport which would allow the navigator to bridge gaps between refugee patients and clinicians postpartum. Future studies could include a qualitative and quantitative evaluation of a pilot postpartum navigator program for refugee women.

A strength of this study is the use of a qualitative research design which allowed for in-depth exploration of postpartum experiences among refugee women. Additionally, this study has diverse sample population that includes women from different countries of origin resettled in different regions of California. There are several limitations to this study as well. First, our study may not be representative of the perspectives of those who are more isolated or less in need of support. This is mainly because our participants were recruited through community organizations that support refugees. Thus, our sample may be bias towards women who are well-informed and connected to support services. Additionally, the perspective represented in our study may not apply to recent arrivals or those who have been resettled in the US for much longer. The average number of years resettled across our sample was 4.5 years. Needs of mothers may be different dependent on the number of years she has been in the US, thus future studies should focus on the experiences and needs of refugees who have been resettled for less than one year. Lastly, most of our participants had a medically uneventful postpartum experience. Patients with postpartum complications may have different support needs.

## Conclusions

This study highlights the need for more tailored postpartum support for refugee women. We demonstrate that postpartum navigation is a welcomed approach to bridging gaps between the patient and the healthcare system postpartum among refugee women. Notably, our study identifies language concordance as an important factor for refugee women who may utilize navigator services. Refugee postpartum women desire a navigator they can trust and communicate with comfortably to obtain information about the healthcare system in the US. The findings of this study underscore the utility of a navigator program in destigmatizing postpartum mental health care, reducing postpartum contraception misconceptions, and providing emotional support.

## Supplementary Information

Additional file 1.

Additional file 2.

## Data Availability

The datasets analyzed during the current study are available from the corresponding author upon reasonable request.

## Abbreviation

US: United States
