# Supplementary material for: Barriers to postpartum health and opinions on a postpartum peer navigator program amongst refugee women resettled in California
Source: BMC Pregnancy Childbirth. 2025 Mar 29;25:372. doi: 10.1186/s12884-025-07479-2 (PMC11954318; doi:10.1186/s12884-025-07479-2)
Supplement: Supplementary file 1 — Additional file 1. [file 12884_2025_7479_MOESM1_ESM.docx]

**Exploring Postpartum Experiences and Interest in a Postpartum Navigator Program for Refugee Communities: Interview Guide**

**The following questions will be asked verbally in the participants preferred language.**

**Demographic Information:**

1. What is your age?
2. What is your country of origin?
3. What is the primary language you speak at home?
4. Do you typically use an interpreter when at a doctor’s appointment?
5. What is your highest level of education completed?
6. What is your marital status?
7. How many years have you been in the US?
8. What type of health insurance do you have?
9. When did you give birth most recently?
10. Are you a first-time mom?
11. What was the mode of your most recent delivery?
12. Where did you give birth most recently? (i.e. home, hospital, birth center, other)
13. Did you breastfeed after your most recent delivery?
14. What medical conditions did you experience during pregnancy? (i.e. diabetes, high blood pressure, other)

**Postpartum Experience:**

1. What was your understanding of what happened during your birth? (ex. Did you know type of delivery you had and why)
2. What was your understanding of how to care for yourself after delivery?

1. What could have helped you better understand your delivery and how to care for yourself?
2. What resources (mental health support, breastfeeding support, help finding a pediatrician) were you provided with after delivery?
3. Tell me about your experience with using any of the resources you were provided?
4. Tell me about the type of social support you had after giving birth? (partner, family, community?)
5. After leaving the hospital postpartum what concerns did you have about your health? Where did you turn to for answers or help?
6. Tell me about your experience with urgent healthcare needs after delivery? (i.e. ED, urgent care)
7. Describe the conversations you had with healthcare providers about birth spacing options after giving birth.
8. Tell me about any discussions you had with a healthcare provider about your mood/mental health after giving birth.
9. How did you feel emotionally after delivery? What helped you cope with your emotions after giving birth?
10. Describe any discussions you had with a healthcare provider about breastfeeding.
11. What was challenging about breastfeeding?
12. Tell me about your 6-week postpartum visit? Why did you decide to attend it/Why not?
13. What was the biggest challenge you encountered after giving birth? What would have helped you overcome this challenge?

**Postpartum Peer Navigator:**

1. What are your thoughts on a program that assigned you to a trained support person during the postpartum period? Would you sign up? Why/why not?
2. Is it important to you that the support person speaks the same language as you? Why/why not?
3. Is it important that the support person is from the same ethnicity as you? Why/why not?
4. How would you like the navigator/guide to engage with you? Come with you to appointments? Visit you in person? Call you once a week?
5. Should this program only be for new moms? Why/why not?
